# A comprehensive analysis of amino-peptidase N1 protein (APN) from Anopheles culicifacies for epitope design using Immuno-informatics models

**DOI:** 10.6026/97320630015600

**Published:** 2019-09-17

**Authors:** Renu Jakhar, Pawan Kumar, Neelam Sehrawat, Surendra Kumar Gakhar

**Affiliations:** 1 Centre for Medical Biotechnology, Maharshi Dayanand University, Rohtak - 124001, Haryana; 2 Centre for Bioinformatics, Maharshi Dayanand University, Rohtak - 124001, Haryana;; 3 Department of Genetics, Maharshi Dayanand University, Rohtak - 124001, Haryana

**Keywords:** Anopheles culicifacies, amino-peptidase N, malaria, epitope, immuno-informatics

## Abstract

Analysis of the Amino-peptidase N (APN) protein from Anopheles culicifacies as a vector based Transmission Blocking Vaccines (TBV) target has been considered for malaria vaccine development. Short peptides as potential epitopes for B cells and cytotoxic T cells and/or helper T cells were identified using prediction models provided by NetCTL and IEDB servers. Antigenicity determination, allergenicity, immunogenicity, epitope conservancy analysis, atomic interaction with HLA allele specific structure models and population coverage were investigated in this study. The analysis of the target protein helped to identify conserved regions as potential epitopes of APN in various Anopheles species. The T cell epitopes like peptides were further analyzed by using molecular docking to check interactions against the allele specific HLA models. Thus, we report the predicted B cell (VDERYRL) and T cell (RRYLATTQF for HLA class I and LKATFTVSI for HLA class II) epitopes like peptides from APN protein of Anopheles culicifacies (Diptera: Culicidae) for further consideration as vaccine candidates subsequent to in vitro and in vivo analysis.

## Background

Malaria continues to remain as a life threatening infectious disease throughout the tropical region of the world. The world malaria report (2018) shows that there are about 219 million cases in 90 countries in the year 2017 alone. Malaria kills more than 600,000 people yearly, mainly children, and eradication is a global priority. India contributes about 4% to total global malaria burden (WHO Report, 2017). Progress has been made in the identification of parasite antigens responsible for transmission-blocking activity [1-3]. Recombinant technologies accelerated evaluation of these antigens as vaccine candidates, and it is possible to induce effective transmission-blocking immunity in humans both by natural infection and now by immunization with recombinant vaccines [4]. Malaria transmission-blocking vaccines are advancing in clinical trials, and strategies for their introduction must be prioritized in favour of the vulnerable populations exposed to the disease [5]. A variety of proteins from Plasmodium falciparum has been previously tested for transmission blocking, however discoveries on the use of multiple mosquito midgut molecules by P. falciparum has diverted the attention of the scientific community towards vector based transmission blocking vaccines [6].

A midgut specific protein, Aminopeptidase N 1 (APN1) is glycosylphospotidyl inositol anchored protein reported to play an important role in ookinete invasion of Plasmodium in the Anopheles gambiae [7]. Aminopeptidase N belongs to a group of membrane bound ubiquitous zinc metallo-proteases (ZMP). Because of the lack of any effective and economical control strategy, TBVs, promise a more efficient way to malaria control. Other studies have shown that the APN protein is a candidate antigen for vaccine development [8]. Studies on the APN 1 gene of Anopheles gambiae have shown it as a potential candidate to induce specific humoral and cellular immunity in BALB/c mice [9]. Structural analysis of midgut APN1 in Anopheles gambiae has revealed B cell epitope based malaria transmission blocking activity [10]. However, T-cell-based epitope mapping is lacking for cellular immunity which is also essential for cleaning parasite infection.

The vaccination aim is to induce immunity against specific pathogens. It will be induced by selectively stimulating antigen specific cytotoxic T-cells, helper T-cells and B-cells. Ideally, a vaccine is divided into two classes based on antigenic epitopes, firstly a B-cell epitope and a helper T-cell epitope, secondly a CTL epitope. The vaccine is capable to induce either specific humoral or cellular immune response against the specific pathogens using combination of these epitopes like peptides [11]. It is of interest to identify conserved regions as epitopes in various species of Anopheles that elicit both neutralizing antibody and cellular immunity against parasite towards the development of an effective transmission blocking vaccine for malaria.

It should be noted that An. culicifacies (Diptera: Culicidae) is an important malarial vector responsible for 60-70 % of cases in India [12]. A comprehensive analysis of amino-peptidase N1 protein (APN) from Anopheles culicifacies for epitope design using Immuno- Informatics models was completed. The data reported here will help identify epitopes to draw strategy for transmission blocking malaria vaccine development.

## Materials and Methods:

### Retrieval of protein sequence from database:

**Figure 1:**
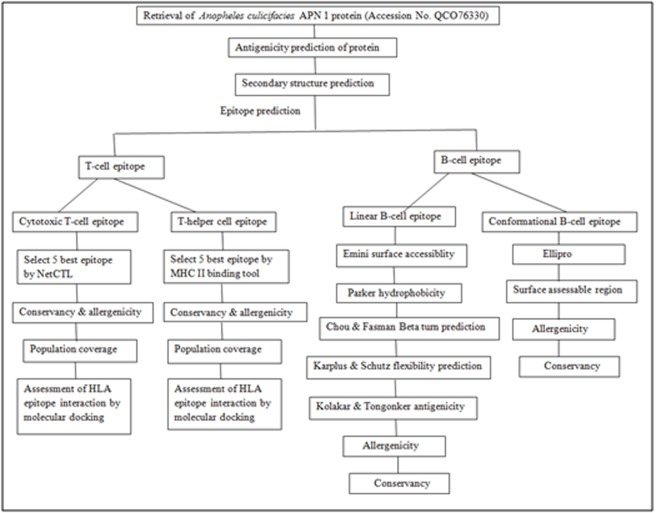
A flowchart representing the methodology applied in the study; arrows represent flow of information and transition from one step to another.

The protein sequence of APN 1 gene (accession no. QCO76330) from An. culicifacies A was downloaded from the NCBI database (Figure 1). The antigenicity of the sequence was predicted using the VaxiJen v2.0 server [13] with default parameters. Further the APN1 protein sequence from different mosquito species (Diptera: Culicidae) were downloaded from the vectorbase database (https://www.vector base.org/). Multiple sequence alignment (MSA) of APN1 protein sequences from these species was completed using Clustal W.

### Secondary structure analysis:

Antigenicity depends on the protein secondary structure. Therefore, prediction of secondary structures using the ExPASy's server ProtParam [14] was completed. Various parameters like the amino acid composition, extinction coefficient, instability index, aliphatic index and molecular weight are included. Self-optimized prediction method (SOPMA) [15] was also used to study transmembrane helices, solvent accessibility, globular and coiled regions for the analysis of secondary structure in the APN1 protein. These methods provided information about the protein stability with potential functional role for APN1.

### Prediction of B cell epitope:

Immune Epitope Database (IEDB) was used to predict B cell epitopes. The tools at the IEDB, Bepipred linear epitope prediction [16], Emini surface accessibility [17], Kolaskar and Tongaonkar antigenicity [18], Parker hydrophobicity [19], Chou and Fasman beta turn prediction [20] and Karplus and Schulz Flexibility Prediction [21] were used in this study. The predicted linear epitopes having equal or more values than average default threshold values are surface accessible, antigenic, hydrophilic and flexible and lie in beta turn regions. ElliPro [22] at IEDB was used to predict conformational B-cell epitopes.

### Prediction of cytotoxic T cell epitopes:

The NetCTL server [23] was used to predict T-cell epitopes in this study. The parameter value was set at 50 to have highest specificity and sensitivity of 0.94 and 0.89, respectively. It should be noted that all available HLA super types were selected for the antigen protein sequence analysis. A combined algorithm of class I HLA-peptide binding, transport efficiency, Transporter of Antigenic Peptide (TAP) and proteosomal cleavage efficiency were considered to conclude scores. The best epitope was selected based on the combined score values.

Putative epitopes were further tested for class I HLA binding using IEDB [24]. Stabilized Matrix Base Method (SMM) was used to calculate the threshold values for strong binding peptides (IC50). Nine residue amino acids length peptide was selected for all the alleles. Alleles having IC50 value less than 200nm were selected for further workflow [25]. Immunogenicity prediction tool at IEDB was used to predict immunogenicity of the epitopes [26].

### Prediction of helper T cell epitope:

Helper T cell (HTL) epitopes were predicted by using HLA II binding tool on IEDB [27]. It covers all HLA class II alleles including HLA-DR, HLA-DP and HLA-DQ [28]. IC50 below 200 nM show maximum interaction potentials of HTL epitope and HLA II allele [29].

### Conserved regions in antigens and allergenicity assessment:

The conserved epitope analysis was carried out in the APN1 protein sequences from fifteen different species of mosquito by analysing conservation across antigens using IEDB [30]. Similarly, the allergenicity of the epitopes was analyzed by the Allertop for evaluation of allergenicity in proteins [31].

### Epitopes three dimensional structures:

Epitopes in three dimensional structures were assigned using PEPFOLD [32].

### Population coverage prediction:

Human population coverage for selected epitopes was checked by population coverage tool at IEDB [33]. Data for epitopes, HLA alleles, ethnic groups and geographical regions across the world were considered.

### Assessment of HLA-peptide interaction using molecular docking:

Molecular docking studies help study epitope binding with HLA molecules [34]. Autodock Vina [35] and and Lig Plot+ [36] was used to analyze the interactions between HLA and epitopes. HLA class I and II 3D structures were downloaded from RCSB PDB [37]. Prior to docking, bound epitope was removed by using Pymol. Three dimensional structures of An. culicifacies protein are modeled by using the protein homology modelling tool Swissmodeler [38]. Energy minimization was done with Chimera [39] and structure validation was carried out with SAVES [40], QMEAN [41] and Prosa [42].

## Results:

### Retrieval of protein sequence and antigenicity determination:

APN1 protein sequence of An. culicifacies retrieved from NCBI in FASTA format was screened using the VaxiJen server to predict immunogenicity. The APN1 (QCO76330) is a known antigenic protein based on overall immunogenicity prediction score.

### Secondary structure analysis:

**Figure 2:**
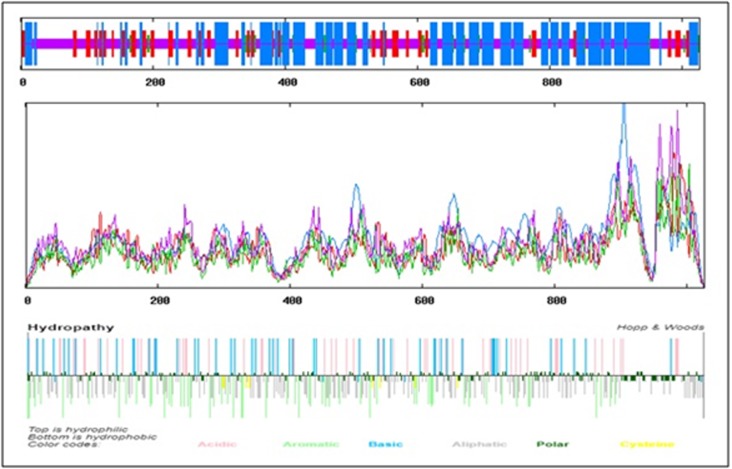
(A) Secondary structure plot of An. culicifacies APN1. Helix is indicated by blue, while extended strands and beta turns are indicated by red and green, respectively.(B) Hopp and Woods Hydropathy plot for An. culicifacies APN1 is shown.

Secondary structure analysis of the APN1 protein (1027 amino acid, molecular weight of 114 kDa, isoelectric point of 5.05, formula of C5115H7902N1356O1551S39) have 445 alpha helixes (43.3%), 147 extended strands (14.31%), 35 beta turns (3.41%) and 400 random coils (38.95%) (Figure 2). Amino acid composition show the presence of alanine (9.9%) and threonine residues (10.5%), suggesting that these residue might be in high biological demand during development. Total number of positively charged residues (Arg + Lys) is 80 and negatively charged residues (Asp + Glu) are 110. The estimated net charge of this protein is -29.2 at pH 7 with poor water solubility.

### B-cell epitope identification:

**Figure 3:**
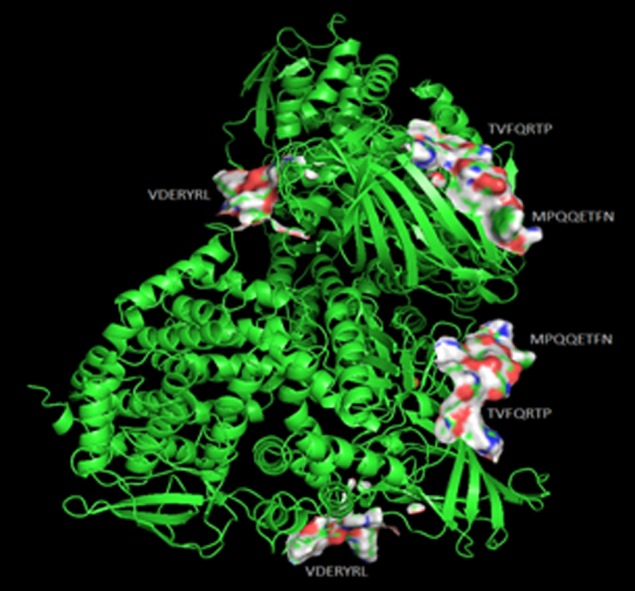
Three dimensional representation of B cell epitopes V_66_DERYRL, T_256_VFQRTP and M_253_PQQETFN showing surface accessibility on both A and B chains of the APN 1 protein is illustrated. These epitopes are present on the surface assessable region of the antigen. Red and white regions are capable of interacting with the nearby residues.

**Figure 4:**
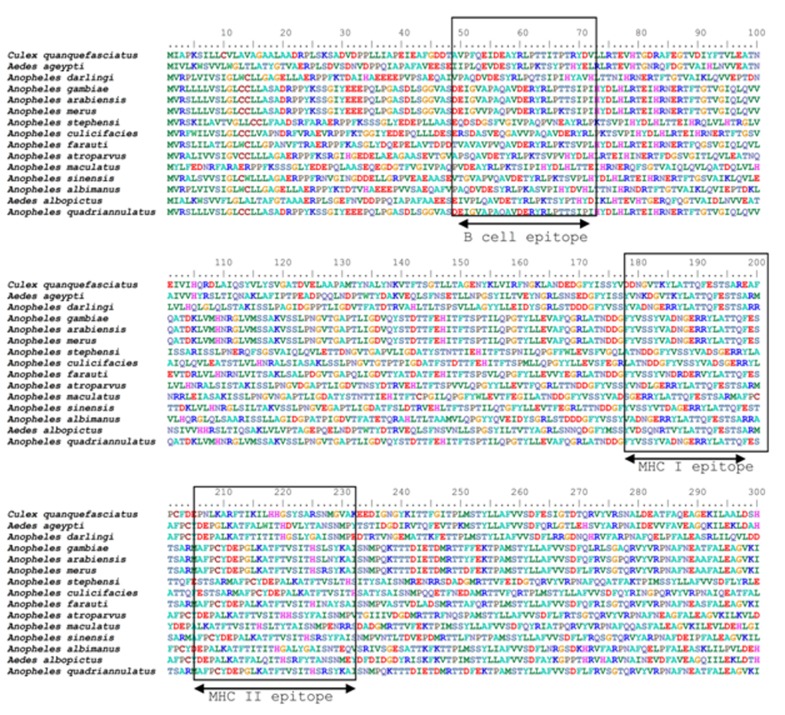
Conserved potential MHC I, MHCII and B cell epitopes with position in the amino acid sequence of APN1 protein in different mosquito species is shown. Mosquito species with respective vectorbase ID used in this analysis are given as follows: An. gambiae (AGAP004809), An. arabiensis (AARA016470), An. merus (AMEM002547), An. farauti (AFAF015666), An. quadriannulatus (AQUA016895), An. sinensis (ASIC009153), An. atroparvus (AATE011993), An. darlingi (ADAC006959), An. maculatus (AMAM007684), An. albimanus (AALB015678), An. culicifacies (QCO76330*), An. stephensi (KJ573522*), Ae. albopictus (AALF017287), Ae. ageypti (AAEL012778), Cu. quanquefasitus (CPIJ001048). *represents NCBI acession no.

Linear B cell epitopes were predicted on the basis of five algorithms- Parker hydrophilicity, Emini surface accessibility, Chou and Fasman beta turn prediction, Kolaskar and Tongaonkar antigenicity and Bepipred linear epitope prediction available on IEDB. All values greater than the average value were considered as potential antigenic determinants. Three epitopes were found to have cutoff prediction scores above threshold scores and nonallergic in nature, namely VDERYRL, MPQQETFN and TVFQRTP (Table 1). These epitopes are found in surface assessable region, their positions on 3D structures and area surface assessable are shown in Figure 3. Among these three epitopes, VDERYRL epitope is conserved in various Anopheles species taken in this study (Figure 4). The conformational B-cell epitopes were also obtained in four chains of APN1 protein by using ElliPro. ElliPro gives the score to each output epitope, which is Protrusion Index (PI) value averaged over each epitope residue. A number of ellipsoids approximated the tertiary structure of the protein. The highest probability of a conformational epitope was calculated at 74% (PI score: 0.74). Residues involved in conformational epitopes, their number, location and scores are also predicted.

### Cytotoxic T-cell epitopes identification:

Epitopes having high combinatorial scores were considered as most potential epitopes as predicted by NetCTL. HLA-I allele interactions with these epitopes were completed using SMM-based IEDB HLA-I binding prediction tool. The epitopes with higher affinity (IC50 less than 200) with MHC-I alleles were selected for further analysis (Table 2). The affinity for binding of the epitopes with the HLA-I alleles was inversely propotional with the IC50 values. The predicted total score of proteasome score, tap score, HLA score, processing score and HLA-I binding are summarized as total score in Table 2. These epitopes are antigenic and nonallergic in nature. Among these five T-cell epitopes, 9-mer epitope, RRYLATTQF was found to have the highest combined score and it interacts with twelve HLA-I alleles. The conservancy analysis of these epitopes indicated that this epitope was found to be 78 % conserve (Figure 4), which was maximum among all epitopes. However, another epitope NLAERTMLI was found to be 56 % conserve and have more number of allelic interactions with good population coverage than other epitopes.

### Helper T-cell epitope identification:

Putative helper T-cell epitope candidates (9-mer sequences) were antigenic and non-allergic in nature showing interactions with numerous HLA-DR alleles (Table 3). The epitope LKATFTVSI was found to have maximum number of allele binding interactions with highest population coverage and 60 % epitope conservancy (Figure 4), which is the maximum among all selected epitopes.

### Population coverage:

**Figure 5:**
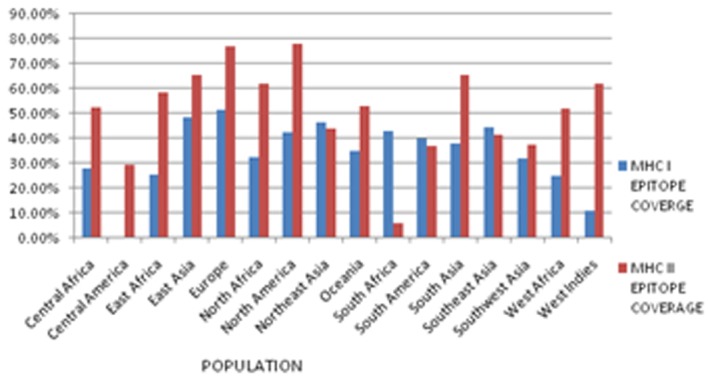
Percentage of population coverage rate for selected HLA I epitope 'RRYLATTQF' and HLA II epitope' LKATFTVSI' in the APN1 protein is shown.

**Figure 6:**
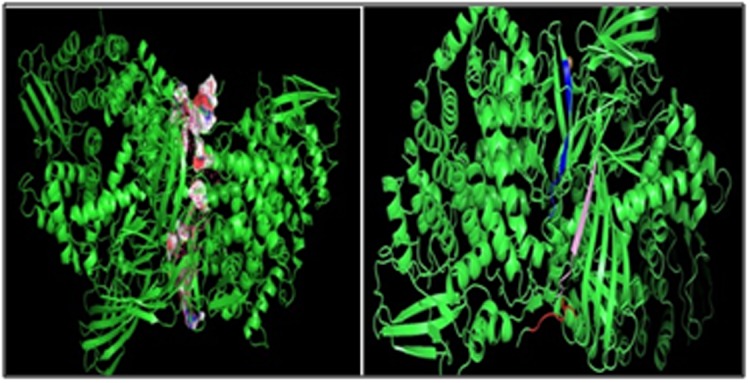
(A) Accessible surface area (ASA) for epitope like peptides in the APN structure model is shown. B and T cell epitopes are shown using red and white colours. Red indicates more compact interaction with the nearby residues. (B) 3D structure representation of the predicted CTL epitope (blue), helper T cell epitope (pink) and B cell epitope (red) of APN1 protein in Anopheles c illustrated by UCSF Chimera visualization tool.

The population coverage of predicted epitopes has been analyzed based on their binding with alleles in sixteen ethnic groups and geographical regions across the world. The high population coverage was found in all putative helper T-cell epitopes and CTL epitopes in 16 geographic regions of the world. The percentage of population coverage rate for selected MHC I epitope 'RRYLATTQF' and MHC II epitope 'LKATFTVSI' of APN1 protein was shown in Figure 5. Also, 3D structure of proposed CTL epitopes, HTL epitopes and B cell epitopes of An. culicifacies APN1 protein illustrated by Pymol (Figure 6). The ASA Plot for APN model over all three epitope residues is also designed. Amino acid interacts with the solvent and the protein core is naturally proportional to the surface area exposed to these environments.

### Docking simulation:

**Figure 7:**
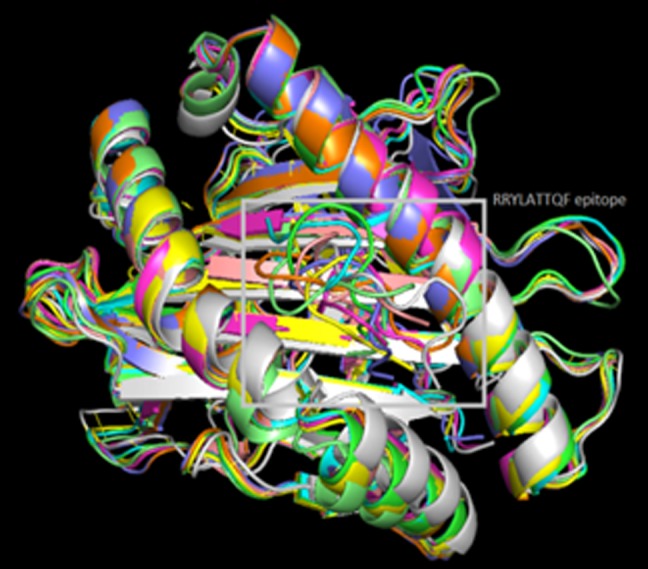
Superposition of the docked predicted peptide (RRYLATTQF) with several class I HLA allele models is shown.

**Figure 8:**
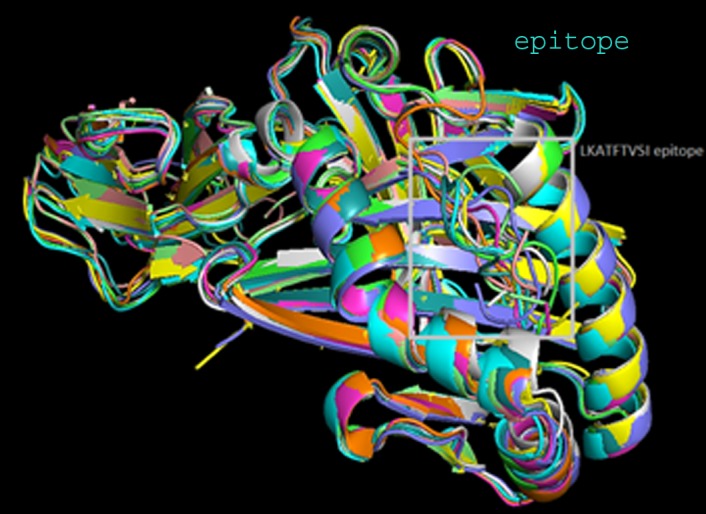
Superposition of the docked predicted peptide (LKATFTVSI) with several class II HLA allele models is shown.

Binding interactions between epitopes and HLA alleles were assessed using Autodock Vina. The 3D structure of epitopes was predicted using PEP-FOLD and energy minimization was carried out by using Yasara. In this study binding of epitope RRYLATTQF were shown with HLA class I alleles. Three-dimensional structures were obtained from RCSB. The receptors used for docking studies included reported HLAs. However epitope (RRYLATTQF) was used as ligand for HLA class I. The grid coordinates from selected receptor molecules for docking with their epitope was selected. 1Å spacing was used to select the binding site. The grid box was positioned carefully to make the docking of ligands at the binding groove of the receptors. The binding energies of predicted epitope with their respective allele's receptor were as shown in Table 4. HLA-C*07:02 was observed to have the best interaction with the RRYLATTQF epitope with lower binding energy (-8.4 Kcal/mol). The predicted peptides showed significant binding affinities with all HLAs (Figure 7). The more negative ΔG binding value, stronger is the interaction between the epitope and HLA. Also, the binding energy of the predicted epitopes were compared with the binding energy of the already experimentally verified peptides and found to be negative. Similarly molecular docking simulation epitope LKATFTVSI were shown with HLA class II alleles (Figure 8). The LKATFTVSI - HLA-DRB1*11:01 complex shows lowest ΔG binding value (-7.9 kcal/mol) among all the complexes (Table 5). Strong binding affinities give strong indicative clear idea that peptide vaccine designed by using these epitopes may efficiently work in vivo to elicit humeral and cell mediated immunity.

## Discussion

Malaria transmission blocking vaccine helps control malaria without causing ecological imbalance. During the present study, the most potent B and T cell epitopes for transmission blocking vaccine in APN1 protein of An. culicifacies based on computational techniques. APN1 was found to be the immunogenic protein by Vaxijen server and this has also been indicated as a lead TBV candidate [5]. The analysis of secondary structure of APN1 revealed that its antigenic part is more likely to be the beta sheet region as also reported in other experiment [40]. The presence of threonine residues (10.5%) predominately in the beta sheet also indicates the protein's antigenicity. The predicted negative value (- 0.096) of grand average of the hydrophobicity rule (GRAVY) of this linear sequence protein not only indicates its hydrophilic nature but also indicates the presence of residues mostly on the surface. In addition, this protein is stable and aliphatic in nature because its Instability Index (33.25) is smaller than 40 and Aliphatic Index (85.53) has higher value. High aliphatic index seems to be responsible for increasing the thermo stability of globular proteins. Also higher proportions of coiled region provide more stability.

B and T cell epitopes involves in humoral and cell mediated immunity. Two types of B cell epitopes are linear epitopes and conformational epitopes. We predicted three linear (continuous) epitopes based on scores which were above threshold values of five algorithms- Parker hydrophilicity, Emini surface accessibility, Chou and Fasman beta turn prediction, Kolaskar and Tongaonkar antigenicity and Bepipred linear epitope prediction available on IEDB. The more value of B cell epitope scores then the threshold level in five algorithms indicates that these candidate epitopes (VDERYRL, MPQQETFN and TVFQRTP) could be effective antigenic peptides in response to B cells. The localization of conformational (discontinuous) epitopes on A and B chain of the APN1 protein using 3D representation of residues revealed that the presumptive antigenic epitopes sequence that is placed in such a way which enables it to have direct interactions with immune receptor. The B-cell epitopes residues, 66VDERYRL72 situated on the surface of B chain of APN1 protein had good Protrusion Index (PI) score (0.738) were indicative of high accessibility. Ellipsoid value of PI 0.73 indicates that 73% protein residues lie within ellipsoid and the remaining 27% residues lie outside. PI score and solvent accessibility are directly proportional to each other, if PI score is higher; maximum is the solvent accessibility of the residues. Thus, these could be the putative vaccine candidates.

T-cell based development of vaccines seems to have potential because of antigenic drift as the foreign particles can easily engineer the escape from antibody memory response. In addition T-cell mediated immunity tends to be a long lasting. The peptide that passes several criteria has been considered to be a good epitope candidate such as possessing antigenicity, non-allergen, highly immunogenic, good conservancy, good interaction with HLA molecules and enough population coverage. During the present study, it was found that the epitope NLAERTMLI could be used as a potential candidate because it had the maximum number of HLA binding alleles amongst other CTL epitopes, but having less conservancy and combined score. This inconsistency of immunological features of epitopes indicates that some other parameters also needed for screening. An epitope should be highly conserved among different species of Anopheles. The conservancy analysis of these epitopes indicated that RRYLATTQF was found to have maximum conservation almost all Anopheles species consider in this study. It also had highest combined score and immunogenicity score than NLAERTMLI. Armistead et al. (2014) have indicated that 135-amino-acid fragment located in 60-195 amino acid sequence of An. gambiae APN1 is safe and highly immunogenic, even in the absence of an adjuvant, in murine models. Interestingly CTL epitope (RRYLATTQF) and B cell epitope (VDERYRL) predicted during the present study coincides with this location.

The maximum number of alleles binding interactions of epitope LKATFTVSI with MHC class II was observed using IEDB server. This epitope was predicted to have maximum conservancy among other epitopes. These epitopes was nonallergic and antigenic in nature. The peptide that fulfills the above said parameters, RRYLATTQF for MHC class LKATFTVSI and I for MHC class II, were further chosen for docking studies. Docking simulation study of the predicted MHC peptides with HLA molecules was performed to find out that whether the designed epitope would elicit the sufficient immunological responses in vivo. The binding energy of predicted MHC I epitope with HLA-B*27:05 recep¬tor was found to be -7.9 kcal/mol as compared to the binding energy of Nipah virus V protein predicted epitope (NPTAVPFTL) with HLA-B*27:05 (-3.13 kcal/mol) and was observed to be lower in the predicted epitope [43]. The interaction between the epitope and HLA are stronger if ΔG-binding value is more negative. The similar results were also found in the molecular dock¬ing simulation between MHC class II-restricted epitope and HLA. The LKATFTVSI- HLA-DRB1*11:01 complex had the lowest binding energy (-7.6 kcal/mol) of all the studied complexes. The strong binding affinity showed that peptide vaccine designed by using these selected epitopes might be well work in vivo to elicit cell mediated and humoral immunity.

Different ethnic populations have high polymorphism in HLA. HLA proteins restrict the reaction to T-cell epitopes. Therefore, to stimulate immune responses in human populations among world, the HLA specificity of T-cell epitopes has to be measured as main criteria for selection of the epitopes. On the basis of above study, the epitope candidates should bind maximum HLA alleles to get better population coverage. In this study, the five HTL and CTL epitopes have shown good population coverage (74% for MHC I and 59% for MHC II in average) and reached above average values in Europe, North America, North Africa and south Asia population. Further analysis has shown that helper T-cell epitopes RRYLATTQF (33%) for MHC class-I and CTL epitope LKATFTVSI (60%) for MHC class-II (that bind the maximum number of HLA alleles) is reported. It should be noted that An. culicifacies is a prominent species in India. NPTAVPFTL for MHC class I show highest population coverage in India. These epitopes have good coverage of population and it may provide a broad immune protection to human beings from different regions of the world. The predicted CTL epitope RRYLATTQF for cellular immunity, HTL epitope LKATFTVSI and B cell epitope VDERYRL for humoral immunity may be synthesized for further in vivo and in vitro assays. These results are based on an analysis of available data on various immune databases. The results of the present study suggest that the predicted epitopes are good candidates for making a peptide vaccine which may initiate an effective immune response in vivo.

## Conclusion

We report the predicted B cell (VDERYRL) and T cell epitopes (RRYLATTQF and LKATFTVSI) from the APN1 protein of Anopheles culicifacies (Diptera: Culicidae) for further consideration as vaccine candidates subsequent to in vitro and in vivo analysis.

## Author contribution:

Renu Jakhar conducted the study, performed the analysis and wrote the manuscript. S.K. Gakhar planned the study and edited the manuscript. Neelam Sehrawat analyzed the data. Pawan Kumar helped with the analysis..

## Figures and Tables

**Table 1:** B cell epitopes with allergenicity predicted using the IEDB tool

Epitopes	Start	End	Length	Emini Surface Accessibility Prediction score /Threshold	Karplus and Schulz Flexibility Prediction score/ Threshold	Chou and Fasman Beta-Turn Prediction score/ Threshold	Kolaskar and Tongaonkar Antigenicity Prediction score / Threshold	Parker Hydrophiliciy Prediction score / Threshold	Allergenicity
				1	0.988	0.954	1.028	1.209	
VDERYRL	64	72	7	3.448	1.009	0.904	1.037	1.629	Non allergen
MPQQETFN	242	249	8	2.78	1.099	0.911	0.967	1.957	Non allergen
TVFQRTP	256	262	7	1.1	1.006	0.924	1.035	1.4	Non allergen

**Table 2:** The percentage conservancy, immunogenicity score, population coverage and total processing score of putative T-cell epitopes interacting with class I HLA alleles.

Epitopes	Position	Combined score	Interaction of MHC-1 allele with an affinity <200 ic50	Conservancy (%)	Immunogenicity	Antigenicity	Allergenicity	Population coverage (%)
TTFEHITFT	150	1.0063	HLA-A*68:03, HLA-C*12:03, HLA-A*32:07, HLA-A*02:50, HLA-B*40:13, HLA-B*27:20 HLA-C*03:03, HLA-A*68:02, HLA-C*06:02, HLA-C*07:01, HLA-C*14:02	22.22	0.39669	Antigenic	Non allergen	41
RRYLATTQF	197	2.1248	HLA-B*27:20, HLA-B*15:03, HLA-A*32:07 HLA-B*27:05, HLA-A*68:23, HLA-B*40:13 HLA-C*03:03, HLA-C*12:03, HLA-A*32:15 HLA-C*14:02, HLA-A*32:01, HLA-C*07:02	77.78	0.10028	Antigenic	Non allergen	36
RPMNWNAAT	437	1.3843	HLA-A*68:23, HLA-B*07:02, HLA-B*42:01, HLA-C*12:03, HLA-A*32:15, HLA-B*27:20 HLA-B*40:13, HLA-C*03:03, HLA-A*32:07	66.67	0.20198	Antigenic	Non allergen	21
RVALNLMTY	661	1.5315	HLA-A*68:23, HLA-A*32:07, HLA-A*80:01 HLA-B*15:17, HLA-C*12:03, HLA-C*03:03 HLA-B*27:20, HLA-A*32:15, HLA-A*32:01 HLA-A*29:02, HLA-A*26:02, HLA-B*40:13	11.11	-0.14072	Antigenic	Non allergen	24
			HLA-A*30:02					
NLAERTMLI	802	1.2078	HLA-A*02:50, HLA-B*27:20, HLA-A*02:02 HLA-A*02:03, HLA-A*32:07, HLA-A*02:12 HLA-A*02:19, HLA-C*07:01, HLA-A*02:11 HLA-A*68:23, HLA-A*02:01, HLA-A*02:06	55.56	0.04571	Antigenic	Non allergen	51
			HLA-C*12:03, HLA-C*03:03, HLA-A*32:15 HLA-A*02:16, HLA-B*40:13, HLA-A*68:02					

**Table 3:** The IC50 value, antigenicity, conservancy, allergenicity and population coverage of putative helper T-cell epitope of APN1 interacting with class II HLA alleles

Epitope	Position in sequence	Interaction of MHC-II alleles having ic50 value less than 200nm	Antigenicity	Conservancy (%)	Allergenicity	Population coverage (%)
DTTFEHITF	149	HLA-DRB1*07:01, HLA-DRB1*03:01, HLA-DRB1*15:01	Antigenic	33	Non allergen	31
LKATFTVSI	222	HLA-DRB1*07:01, HLA-DRB1*01:01, HLA-DRB1*04:05, HLA-DRB1*04:01, HLA-DRB1*13:02, HLA-DRB1*11:01, HLA-DRB1*04:04, HLA-DRB5*01:01, HLA-DRB1*15:01, HLA-DRB4*01:01, HLA-DRB3*01:01, HLA-DRB1*12:01	Antigenic	60	Non allergen	51
LSYFNSRLR	685	HLA-DRB5*01:01, HLA-DRB1*04:04, HLA-DRB1*15:01, HLA-DRB1*11:01, HLA-DRB1*04:01, HLA-DRB1*07:01, HLA-DRB4*01:01, HLA-DRB1*04:05, HLA-DRB1*12:01, HLA-DRB1*03:01	Antigenic	40	Non allergen	51
LTTALGSGT	825	HLA-DRB1*01:01, HLA-DRB1*07:01, HLA-DRB1*11:01, HLA-DRB1*04:04, HLA-DRB1*04:05, HLA-DRB5*01:01, HLA-DRB1*15:01, HLA-DRB4*01:01, HLA-DRB1*03:01, HLA-DRB1*12:01	Antigenic	40	Non allergen	52
FEGLMLSNF	938	HLA-DRB1*01:01, HLA-DRB1*04:05, HLA-DRB1*04:04, HLA-DRB1*04:01, HLA-DRB4*01:01, HLA-DRB5*01:01, HLA-DRB1*11:01, HLA-DRB3*01:01, HLA-DRB1*12:01	Antigenic	33	Non allergen	31

**Table 4:** Molecular docking data for class I HLA alleles binding with known epitopes using autodock vina

Protein Name	PDB Id.	Axis	Center Box	Size	Binding Energy (KCal/mol)
HLA-A*68:23	6EI2	X	55.012	40	-8.2
		Y	50.512	46	
		Z	9.289	40	
HLA-B*15:03	5TXS	X	2.957	52	-8
		Y	15.238	40	
		Z	144.516	36	
HLA-B*27:05	1HSA	X	2.986	40	-7.9
		Y	-21.57	40	
		Z	2.564	34	
HLA-B*40:13	5IEH	X	2.858	54	-8.1
		Y	-17.595	40	
		Z	-32.635	40	
HLA-C*03:03	1EFX	X	6.847	44	-8.1
		Y	28.601	30	
		Z	76.272	34	
HLA-C*07:02	5VGE	X	22.237	48	-8.4
		Y	-58.037	40	
		Z	12.009	30	

**Table 5:** Molecular docking data for class II HLA alleles binding with known epitopes using autodock vina

Protein Name	PDB Id	Axis	Center Box	Size	Binding Energy (KCal/mol)
HLA-DRB1*01:01	1AQD	X	8.079	60	-7.6
		Y	22.471	40	
		Z	37.748	54	
HLA-DRB1*04:01	1D5M	X	19.299	50	-7.7
		Y	27.736	54	
		Z	16.637	52	
HLA-DRB1*11:01	6CPM	X	-8.958	44	-7.9
		Y	-17.417	44	
		Z	14.627	52	
HLA-DRB1*15:01	1BX2	X	48.747	48	-7.4
		Y	-4.999	54	
		Z	151.889	50	
HLA-DRB3*01:01	3C5J	X	81.567	56	-7.5
		Y	26.926	50	
		Z	20.078	46	
HLA-DRB5*01:01	1FV1	X	19.669	58	-7.7
		Y	21.027	40	
		Z	2.783	52
